# Sex-specific associations among infant food and atopic sensitizations and infant neurodevelopment

**DOI:** 10.3389/fped.2022.734428

**Published:** 2022-09-06

**Authors:** Nicole Rodriguez, Carmen A. Tessier, Piushkumar J. Mandhane, Jacqueline Pei, Elinor Simons, Theo J. Moraes, Stuart E. Turvey, Padmaja Subbarao, Anita L. Kozyrskyj

**Affiliations:** ^1^Department of Pediatrics, Faculty of Medicine and Dentistry, University of Alberta, Edmonton, AB, Canada; ^2^Department of Educational Psychology, Faculty of Education, University of Alberta, Edmonton, AB, Canada; ^3^Department of Pediatrics and Child Health, University of Manitoba and Children's Hospital Research Institute of Manitoba, Winnipeg, MB, Canada; ^4^Department of Pediatrics, Faculty of Medicine, University of Toronto and Hospital for Sick Children, Toronto, ON, Canada; ^5^Department of Pediatrics, Faculty of Medicine, University of British Columbia, Vancouver, BC, Canada; ^6^Department of Obstetrics and Gynecology, School of Public Health, University of Alberta, Edmonton, AB, Canada

**Keywords:** food sensitization, atopic sensitization, atopy, infant, neurodevelopment, social- emotional infant sensitizations and socio-emotional development

## Abstract

**Introduction:**

Food sensitization is a first and strong indicator of immune deviation in the progression to other allergic conditions. Sensitization to food or other allergens and related inflammation during critical windows of infant development may adversely affect neurodevelopmental milestones. However, additional research is needed to test this association further.

**Methods:**

Associations between atopic (any food or aeroallergen) or food sensitization (specific to egg, soybean, peanut, and milk) at age 1 year and neurodevelopment up to 2 years of age were evaluated in the national CHILD Cohort Study, with a secondary aim examining whether these associations were sex-specific. Food and atopic sensitization were assessed by skin prick tests (SPT) in 1-year-old infants, with neurodevelopment assessed using the cognitive, language, motor, and social-emotional subscales of the Bayley Scales of Infant Development (BSID-III) administered at 1 and 2 years of age.

**Results:**

Atopic sensitization was present among 16.4% of infants, while 13.4% had food sensitizations. Only socioemotional scores reached statistical significance among the four BSID-III domains. Both atopic and food sensitization at 1 year of age was associated with lower social-emotional scores, independent of the infant's ethnicity. These findings were sex-specific and only observed among boys, among whom social-emotional scores were lowered by 5 points if atopic sensitization was present (−5.22 [95% CI: −9.96, −0.47], *p* = 0.03) or if food sensitization was present (−4.85 [95% CI: −9.82,0.11], *p* = 0.06). Similar results were observed using the standard SPT cut-off of ≥3 mm — for atopic sensitization (−5.17 [95% CI: −11.14, −0.80], *p* = 0.09) and for food sensitization (−4.61 [95% CI: −10.96, 1.74], *p* = 0.15).

**Conclusion:**

In our study of term infants, we found an inverse, cross-sectional association between atopic and food sensitization status and social-emotional development scores in male children but not female children.

## Introduction

Food allergy in high-income countries is on the rise, with an allergy to common foods reported in more than 10% of 1-year-old infants (1). Food allergy is a classic Type 1 hypersensitivity response that develops over time when repeated exposure to a food antigen activates allergen-specific T cells. These T cells provide “help” to B cells to secrete allergen-specific IgE (immunoglobulin E), which then primes mast cells to degranulate on subsequent exposure to the allergen, releasing histamine and other inflammatory mediators (2). Mast cells can cause neuroinflammation, and children with high levels of mast cells have a greater risk of developing autism spectrum disorder (ASD) (3).

The phenomenon of the “atopic march,” wherein allergic diseases happen in progression, begins in childhood (4). Food sensitization, determined by skin prick testing or serum IgE levels to the allergen, affects up to 28% of preschool children in the United States (5). While it may not develop into clinically significant food allergy, food sensitization is a first and strong indicator of immune deviation in the atopic march (5, 6). The resultant IgE-mediated immune response to the allergen initiates the inflammatory process resulting in food sensitization (2). Conversely, if the food allergen is blocked by serum or intestinal immunoglobulins (such as IgA), the immune response will be diminished, and tolerance to the allergen will occur. Disease severity is related to the amount of circulating IgE. Among the various food allergens, infant sensitization to peanuts is the most likely to persist into later childhood and/or proceed to food allergy (7, 8). The infant's 1st year of life is the “window of opportunity,” wherein nutrition and other exposures can significantly impact an infant's developing immune and nervous systems (9).

Atopy and impaired neurodevelopment have immune dysregulation and inflammation in common and share many risk factors (10). Accumulating epidemiologic evidence further supports a connection between the infant's immune system and neurodevelopmental disorders. In the comprehensive review by Jyonouchi et al. (11), it was pointed out that allergic symptoms commonly worsen behavioral symptoms of ASD. A newer study of school children revealed that peanut sensitivity or allergic rhinitis in 6-year-olds predicted symptoms of attention-deficit hyperactivity disorder (ADHD) at 12 years of age (12). Temporal associations between atopic disease and neurodevelopment have been also found in very young children with a family history of atopy, whereby 12-month-old infants with any atopic disease (eczema or food allergy) exhibited lower motor scores on the Bayley Scales of Infant Toddler Development at 18 months (13). Among infants with diagnosed food allergy at 12 months, lower social-emotional scores were reported at 18 months (13). Recent findings from the Boston Birth Cohort have demonstrated a higher incidence of neurodevelopmental disabilities among children with atopic disease compared to children without atopy (14).

In this study, we determined the association between food or any allergen sensitization as a marker of IgE dysregulation in the 1st year of life and children's neurodevelopment at the toddler age in a general (not high atopy risk) population. Sex-specific associations were tested. We hypothesized that atopic sensitization adversely affects the neurodevelopment of the growing infant and thus will lead to lower neurodevelopmental scores.

## Methods

### Study design

Our present study accessed data from a subsample of the CHILD birth cohort (www.childstudy.ca), consisting of 537 infants from the Edmonton site. The CHILD birth cohort recruited pregnant women aged ≥18 years who delivered singleton infants at ≥35 weeks of gestational age and birth weight of ≥2,500 g. Multiple gestations, *in vitro* fertilized births, and preterm births were excluded, as were children born with congenital abnormalities or respiratory distress syndrome. Mothers were followed throughout pregnancy, atopic and food sensitizations were both assessed at 1 year of infant age, and infant neurodevelopmental scores at ages 1 and 2 years. Study covariates were collected from study questionnaires during pregnancy and 3 months post-partum and/or hospital birth records. They consisted of the following maternal factors: maternal ethnicity (White Caucasian, Asian, and Other), maternal age (18 to 29, 30 to 39, and over 40), maternal education (some/finished high school, some university/college, university degree), asthma treatment during pregnancy (yes or no), prenatal smoking (yes or no), and maternal depression (never, prenatal, postnatal, and persistent). Maternal diet was also included and was based on the prenatal fruit intake (a “5-a-day” method), which measures the sum of “servings of fruit, not including juices, “plus servings of juice” per day (15). In the CHILD Cohort Study, fruit intake was associated with infant cognition and was based on the 5-day method from a modified 174-item, self-reported Food Frequency Questionnaire (15, 16). Studied infant factors included child sex (male or female), gestational age (in weeks), presence of older siblings (yes or no), birth mode [Vaginal-no IAP (intrapartum antibiotic prophylaxis), Vaginal-IAP, CS-elective, CS-emergency], breastfeeding at 3 months (exclusive breastfeeding, partial breastfeeding, zero, and unknown), and introduction of solid foods at 3 months of infant age (yes or no).

**Figure 1:**
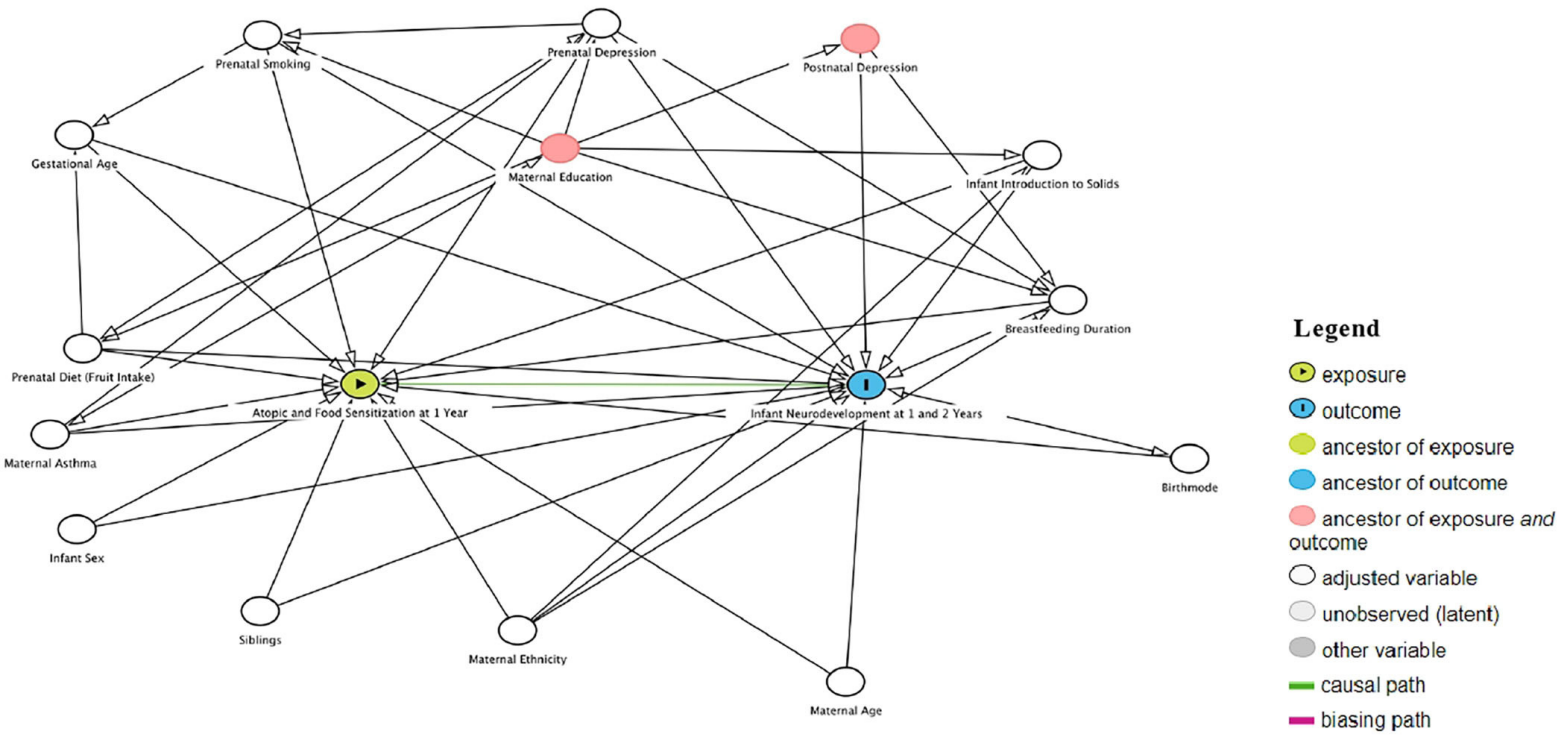
Direct acyclic graph (DAG) representing exposure, covariate, and outcome direct associations to select pontiential confounding factor.

A Direct Acyclic Graph (DAG) approach was pursued to select a minimal adjustment set of potential confounding factors to test further associations between infant sensitization and child Neurodevelopment (Figure 1; (17)). A DAG *gold-standard change-in-estimate* procedure was used where covariates were selected by backward elimination from the initial DAG-adjusted model (18). The Human Research Ethics Board at the University of Alberta approved this study (Ethics No. Pro00103296).

### Food and atopic sensitization assessments

Measures for sensitization were outlined in a previous CHILD Cohort Study paper (19). In our sample, food sensitization was defined as any positive skin prick test to peanut, milk, egg, or soybean allergens. On the other hand, atopic sensitization was defined as any positive skin prick test to test food or aeroallergens. Data from food and atopic sensitization were obtained at 1 year of infant age through a skin prick test (SPT) performed by trained staff with one point of a plastic, bifurcated needle (a Duotip II device by Lincoln Diagnostics), held at a 45° angle with the skin plane after complete submersion into an allergen solution. Ten standardized and common food allergens (peanut, soybean, egg white, and cow's milk) and aeroallergens (*Alternaria tenuis*, cat hair, dog epithelium, *Dermatophagoides pteronyssinus, Dermatophagoides farinae*, German cockroach), a positive control (histamine), and a negative control (glycerin) were tested. As implemented in large-scale infant studies, a wheal size of ≥2 mm in diameter in response to any allergen was considered to indicate positive sensitization for that particular allergen (20). The standard skin prick test procedure that uses a cut-off wheal size ≥3 mm in diameter (21) was also tested in sensitivity analyses.

### Neurodevelopmental assessments

Infant neurodevelopmental scores were obtained from the Bayley Scale of Infant Development Third Edition (BSID-III) at 1 year and 2 years of age. Since BSID-III score assessments are unique to the CHILD Cohort Study's Edmonton site, only infants from this location are included in this study. These scores are a validated and objective measure of a child's neurodevelopment, including cognitive, language, motor development, and social-emotional domains (22, 23). Primary caregivers completed BSID-III questionnaires before the child's bedtime. The Cognitive scale (91 items) assesses visual preference, attention, memory, exploration, manipulation, and concept formation. The Language scale assesses receptive communication (49 items) and expressive communication (48 items). The Motor scale assesses gross motor (72 items) and fine motor (66 items) skills. The Cognitive (0.91), Language (0.93), and Motor (0.92) subscales have high-reliability coefficients, and good test-retest stability with coefficients around 0.80 (22, 23). The Social-Emotional scale (35 items) measures six functional and emotional development milestones that are subdivided into different age groups (24). A registered psychologist trained research staff to administer the SID-III instrument and conducted semi-annual assessments. All scores were obtained based on the child's chronological age at testing. Raw scores were converted to scaled scores, and then to composite scores. The standardized population means for the composite score is 100 (standard deviation of 15). A higher score on the BSID-III scales indicates better abilities.

### Statistical analysis

The distribution of 1-year atopic and food sensitization status and BSID-III scores across study covariates was determined. Comparisons of categorical variables were made using the chi-square or Fisher's exact tests. Continuous variables were compared with the *t*-test if binary and one-way ANOVA if more than two categories. ANOVA was also used to compare the means of neurodevelopmental scores from BSID-III subscales by atopic and food sensitization status. Univariable and multivariable linear regressions were conducted to quantify (*via* beta-coefficients) the association between atopic or food sensitization status and neurodevelopmental scores. Potential confounding factors selected by the DAG approach were retained in multivariable regression models if they met the criterion of 15% change in an estimate for atopic or food sensitization. From the minimum DAG set of maternal ethnicity, age, asthma, prenatal depression, prenatal smoking and prenatal diet, infant sex, gestational age, older siblingship, birth mode, breastfeeding duration, and introduction of solid foods, the following were variables that met the 10% change in estimate criterion and were retained in fully-adjusted models: maternal ethnicity, prenatal diet, prenatal depression, introduction to solids, birth mode, gestational age, and breastfeeding duration (Figure 1). Each multivariable model had a unique minimal set of adjustments based on the DAG and a 15% backward selection model-building approach (Supplementary Table S1). Additionally, we have assessed the normality of all BSID-III scores (see attached Supplementary Figures S1, S2).

## Results

### Participant characteristics

In our study of 537 infants, 52.% were male, and 56.5% had an older sibling (Supplementary Table S2). The mean gestational age was 39 weeks (mean =39.1, SD = 1.4). The majority of their mothers were of Caucasian ethnicity (78.8%), between the ages of 30 and 39 years (68.2%), did not smoke (96.3%), and completed university (55.5%). Most infants were born vaginally, in the absence (52.1%) or presence (23.9%) of intrapartum antibiotic prophylaxis (IAP). At 3 months of age, 58.1% were exclusively breastfed, 27.2% were partially breastfed (breastmilk and formula), and 14.3% were not breastfed; almost all the infants (97.6%) were not yet introduced to solid foods. At 1 year of age, atopic sensitization (to food or aeroallergens) was present in 16.4% of infants; the prevalence of food sensitization was 13.4%, making food the predominant allergen for sensitization at this age.

Atopic or food sensitization was more likely among infants of mothers with a university education (*p* < 0.05) or Asian ethnicity (*p* < 0.001, Supplementary Table S4). At age 1 year, language and motor scores increased by one point for each week of gestational age; infants of Asian ethnicity had the lowest scores on the cognition, language, and social-emotional domains, and boys had the lowest language scores (Supplementary Table S5). In addition to gestational age and Asian ethnicity, several more covariates were associated with lower BSID scores at age 2 years, including male sex (all domains), pre/postnatal maternal depression (social-emotional), a lack of breastfeeding (cognitive/language), and absence of siblings (motor, Supplementary Table S6).

### Assessing the association of infant atopic and food sensitization at 1 year with neurodevelopment at 1 year and 2 years of age

Mean scores from four neurodevelopmental domains at 1 and 2 years were compared between atopy status. We found no statistically significant differences between atopic or food sensitization at age 1 year and BSID-III cognitive, language, or motor scores at age 1 year (Table 1). However, the crude linear regression comparison indicated an inverse association between one-year social-emotional scores and atopic sensitization (4.6 points lower, *p* = 0.01) or food sensitization (4.5 points lower, *p* = 0.01). In male infants (Table 1), these crude differences were of a greater magnitude for atopic sensitization (98. vs. 104.1, *p* = 0.005) and for food sensitization (98.1 vs. 104., *p* = 0.01). No sensitization differences in BSID-III mean scores were seen among female infants at 1 year of age. There were no statistically significant crude differences in mean scores between any of the 2-year BSID subscale domains and atopic or food sensitization in 1-year-old infants in total or in sex-specific strata (Tables 1, 2). Although lower 2-year social-emotional scores were observed for food sensitization in female infants (mean, 105.89 vs. 110.75, *p* = 0.1, Table 2), this was not statistically significant.

**Table 1:** Comparison of mean scores for neurodevelopmental domains at age 1 and 2 years across atopic and food sensitization status at 1 year, all infants and stratified by infant sex.

**All infants**								
	**Atopic sensitization 1YR – YES *N* = 88 (16.39% overall)**	**Atopic sensitization at 1YR – NO *N* = 449** **(83.61% overall)**	***p*–value**	**Food sensitization 1YR– YES *N* = 72** **(13.41% overall)**	**Food sensitization at 1YR– NO *N* = 465 (86.59% overall)**	***p*–value**		
	**Mean (SD)**	**Mean (SD)**		**Mean (SD)**	**Mean (SD)**			
***Infant neurodevelopment 1YR***								
BSID–III cognitive 1 year	109.32 (10.89)	110.18 (10.19)	0.472	108.89 (10.98)	110.22 (10.19)	0.308		
BSID–III language 1 year	107.37 (9.89)	107.95 (12.34)	0.679	107.75 (9.48)	107.87 (12.32)	0.935		
BSID–III motor 1 year	101.58 (12.28)	102.80 (15.08)	0.474	101.36 (12.42)	102.80 (14.97)	0.440		
BSID–III social–emotional 1 year	98.76 (10.94)	103.36 (14.23)	**0.005**	98.71 (11.15)	103.21 (14.13)	**0.011**		
***Infant neurodevelopment 2YR***								
BSID-III cognitive 2 year	105.51 (14.24)	105.80 (14.37)	0.865	105.76 (14.72)	105.74 (14.29)	0.992		
BSID-III language 2 year	99.80 (12.40)	100.24 (11.97)	0.752	99.89 (12.26)	100.21 (12.01)	0.834		
BSID-III motor 2 year	98.74 (9.33)	98.94 (9.53)	0.856	99.01 (9.73)	98.89 (9.46)	0.918		
BSID-III social-emotional 2 year	106.72 (14.74)	109.08 (15.90)	0.202	106.69 (14.97)	109.00 (15.84)	0.250		
**Sex stratified**								
	**Female infants**				**Male infants**			
	**Atopic sensitization** **1YR – YES** ***N** = **N** =* **40 (7.45% among all infants)**	**Atopic sensitization at** **1YR – NO** ***N =* 218 (40.60% among all infants)**	**Food sensitization** **1YR – YES** ***N =* 28 (5.21% among all infants)**	**Food sensitization at** **1YR – NO** ***N =* 230 (42.83% among all infants)**	**Atopic sensitization** **1YR – YES** ***N =* 48 (8.94% among all infants)**	**Atopic sensitization at** **1YR – NO** ***N =* 231 (43.02% among all infants)**	**Food sensitization** **1YR – YES** ***N =* 235 (43.76 % among all infants)**	**Food sensitization at** **1YR – NO** ***N =* 44 (8.19% among all infants)**
	**Mean (SD)**	**Mean (SD)**	**Mean (SD)**	**Mean (SD)**	**Mean (SD)**	**Mean (SD)**	**Mean (SD)**	**Mean (SD)**
***Infant neurodevelopment 1YR***								
BSID-III cognitive 1 year	109.88 (7.72)	110.75 (10.14)	109.64 (7.06)	110.73 (10.9)	98.04 (11.38)	104.12 (14.62)	108.41 (12.93)	109.72 (10.29)
BSID-III language 1 year	107.78 (9.05)	110.13 (12.01)	108.82 (7.07)	109.88 (12.06)	107.02 (10.63)	105.89 (12.32)	107.05 (10.78)	105.91 (12.27)
BSID-III motor 1 year	100.15 (11.39)	103.66 (15.63)	99.29 (11.54)	103.58 (15.42)	102.77 (12.97)	12.00 (14.53)	102.68 (12.90)	102.03 (14.52)
BSID-III social-emotional 1 year	99.62 (10.47)	102.54 (13.78)	99.64 (10.09)	102.39 (13.69)	98.04 (11.38)	**104.12** (14.62)	98.10 (11.89)	**104.00** (14.52)
***Infant neurodevelopment 2YR***								
BSID-III cognitive 2 year	107.88 (12.90)	108.42 (15.24)	108.57 (12.83)	108.30 (15.13)	103.54 (15.12)	103.32 (13.06)	103.98 (15.69)	103.24 (12.97)
BSID-III language 2 year	101.50 (10.71)	103.76 (11.49)	101.18 (9.21)	103.69 (11.61)	98.38 (13.58)	96.93 (11.48)	99.07 (13.90)	96.82 (11.43)
BSID-III motor 2 year	99.45 (9.12)	99.9 (9.53)	99.79 (9.62)	99.88 (9.46)	98.15 (9.55)	97.99 (9.45)	98.52 (9.88)	97.92 (9.39)
BSID-III social-emotional 2 year	107.38 (11.71)	110.74 (15.69)	105.89 (11.14)	110.75 (15.53)	106.17 (17.01)	107.49 (15.98)	107.21 (17.12)	107.27 (15.99)

SD, standard deviation; Statistical comparison of means completed by ANOVA. ^*a*^Bold values are statistically significant.

**Table 2:** Univariate and multivariate linear regression for sensitization at 1 year vs. social-emotional scores at 1 and 2 years, all infants and stratified by infant sex (*N* = 537).

**Atopic sensitization multivariate model adjustments - all infants**				
**BSID - III scores at 1YR**	**Crude estimate (95% CI)**	***p*-value**	**Fully-adjusted model estimate (95% CI)**	***p-*value**
Cognitive 1-year	−0.86 (−3.22, 1.50)	0.47	0.441 (−2.17, 3.05)	0.74
Language 1-year	−0.58 (−3.34, 2.18)	0.68	0.64 (– 2.25, 3.54)	0.66
Motor 1-year	−1.22 (−4.58, 2.13)	0.47	−1.93 (−5.06, 1.20)	0.23
Social–emotional 1-year	−4.59 (−7.80, −1.39)	**0.01**	−4.13 (−7.41, −0.86)	**0.01**
**BSID – III scores at 2YR**	**Crude estimate (95% CI)**	***p*–value**	**Fully–adjusted model estimate (95% CI)**	***p*** **–value**
Cognitive 2-year	−0.28 (−3.57, 3.00)	0.87	1.99 (−1.74, 5.72)	0.30
Language 2-year	−0.44 (−3.20, 2.31)	0.75	2.17 (−0.78, 5.11)	0.15
Motor 2-year	−0.20 (−2.38, 1.97)	0.86	0.89 (−1.49, 3.27)	0.24
Social–emotional 2-year	−2.36 (−5.98, 1.27)	0.20	−0.61 (−4.43, 3.21)	0.76
**Food sensitization multivariate model adjustments – all infants**				
**BSID – III scores at 1YR**	**Crude estimate (95% CI)**	***p*–value**	**Fully–adjusted model estimate (95% CI)**	***p*–value**
Cognitive 1-year	−1.33 (−3.89, 1.23)	0.31	−0.27 (−2.90, 2.36)	0.84
Language 1-year	−0.12 (−3.12, 2.87)	0.94	0.76 (−2.36, 3.89)	0.63
Motor 1-year	−1.43 (−5.08, 2.21)	0.44	−2.37 (−5.77, 1.03)	0.17
Social–emotional 1-year	−4.50 (−7.97, −1.02)	**0.01**	−4.01 (−7.55, −0.48)	**0.03**
**BSID – III scores at 2YR**	**Crude estimate (95% CI)**	***p*–value**	**Fully–adjusted model estimate (95% CI)**	***p*–value**
Cognitive 2-year	0.02 (−3.55, 3.59)	0.99	2.28 (−1.74, 6.30)	0.27
Language 2-year	−0.32 (−3.32, 2.68)	0.83	2.19 (−1.00, 5.38)	0.18
Motor 2-year	0.12 (−2.24, 1.49)	0.92	1.25 (– 1.33, 3.83)	0.34
Social–emotional 2-year	−2.31 (−6.25, 1.63)	0.25	−0.59 (−4.72, 3.54)	0.78
**Atopic sensitization multivariate model adjustments – male infants**				
**BSID – III scores at 1YR**	**Crude estimate (95% CI)**	***p–*** **value**	**Fully–adjusted model estimate (95% CI)**	***p*** **–value**
Cognitive 1-year	−0.80 (−4.15, 2.56)	0.64	0.80 (−3.06, 4.66)	0.68
Language 1-year	1.13 (−2.67, 4.93)	0.56	2.49 (−1.68, 6.65)	0.24
Motor 1-year	0.37 (−3.73, 4.48)	0.73	−0.02 (−4.17, 4.13)	0.99
Social–emotional 1-year	−6.08 (−10.58, −1.58)	**0.01**	−5.22 (−9.96, −0.47)	**0.03**
**BSID – III scores at 2YR**	**Crude estimate (95% CI)**	***p–*value**	**Fully–adjusted model estimate (95% CI)**	***p*–value**
Cognitive 2-year	0.22 (−3.97, 4.42)	0.92	4.53 (−0.49, 9.54)	0.08
Language 2-year	1.45 (−2.26, 5.15)	0.44	5.40 (1.22, 9.58)	**0.01**
Motor 2-year	0.15 (−2.80, 3.11)	0.92	1.61 (−1.83, 5.04)	0.36
Social–emotional 2-year	−1.32 (−6.42, 3.78)	0.61	1.79 (−3.95, 7.52)	0.54
**Food sensitization multivariate model adjustments – male infants**				
**BSID – III scores at 1YR**	**Crude estimate (95% CI)**	***p*–value**	**Fully–adjusted model estimate (95% CI)**	***p*–value**
Cognitive 1-year	−1.31 (−4.78, 2.16)	0.46	1.15 (−2.59, 4.89)	0.55
Language 1-year	1.14 (−2.80, 5.08)	0.57	2.30 (−2.08, 6.68)	0.30
Motor 1-year	0.26 (−4.00, 4.51)	0.78	−0.19 (−4.48, 4.11)	0.93
Social–emotional 1-year	−5.91 (−10.59, – 1.23)	**0.01**	−4.85 (−9.82, −0.11)	0.06
**BSID – III scores at 2YR**	**Crude estimate (95% CI)**	***p*–value**	**Fully–adjusted model estimate (95% CI)**	***p*–value**
Cognitive 2-year	0.73 (−3.61, 5.08)	0.74	5.53 (0.34, 10.72)	**0.04**
Language 2-year	2.25 (−1.58, 6.08)	0.25	6.54 (2.23, 10.85)	**0.00**
Motor 2-year	0.60 (−2.46, 3.66)	0.70	2.25 (−1.32, 5.82)	0.22
Social–emotional 2-year	−0.06 (−5.35, 5.23)	0.98	3.57 (−2.39, 9.53)	0.24
**Atopic sensitization multivariate model adjustments– female infants**				
**BSID – III scores at 1YR**	**Crude estimate (95% CI)**	***p*–value**	**Fully–adjusted model estimate (95% CI)**	***p*–value**
Cognitive 1-year	−0.87 (−4.20, 2.45)	0.61	−0.15 (−3.78, 3.48)	0.94
Language 1-year	−2.35 (−6.29, 1.58)	0.24	−1.40 (−5.43, 2.62)	0.49
Motor 1-year	−3.92 (−8.67, 0.83)	0.18	−4.36 (−9.22, 0.50)	0.08
Social–emotional 1-year	−2.92 (−7.50, 1.66)	0.21	−2.99 (−7.57, 1.58)	0.20
**BSID – III scores at 2YR**	**Crude estimate (95% CI)**	***p*–value**	**Fully–adjusted model estimate (95% CI)**	***p*–value**
Cognitive 2-year	−0.54 (−5.59, 4.51)	0.83	−0.82 (−6.34, 4.71)	0.77
Language 2-year	−2.26 (−6.12, 1.59)	0.25	−1.17 (−5.09, 2.75)	0.56
Motor 2-year	−0.49 (−3.70, 2.71)	0.76	0.09 (−3.34, 3.51)	0.96
Social–emotional 2-year	−3.37 (−8.51, 1.77)	0.20	−1.96 (−7.28, 3.37)	0.47
**Food sensitization multivariate model adjustments – female infants**				
**BSID – III scores at 1YR**	**Crude estimate (95% CI)**	***p*–value**	**Fully–adjusted model estimate (95% CI)**	***p–*value**
Cognitive 1-year	−1.09 (−4.95, 2.78)	0.58	−1.36 (−5.29, 2.57)	0.50
Language 1-year	−1.06 (−5.64, 3.53)	0.65	−0.70 (−5.32, 3.92)	0.77
Motor 1-year	−4.68 (−10.21, 0.85)	0.16	−5.54 (−11.15, 0.07)	0.05
Social–emotional 1-year	−2.74 (−8.02, 2.53)	0.31	−2.82 (−8.07, 2.43)	0.29
**BSID – III scores at 2YR**	**Crude estimate (95% CI)**	***p*–value**	**Fully–adjusted model estimate (95% CI)**	***p*–value**
Cognitive 2-year	0.27 (−5.61, 6.40)	0.93	−1.06 (−7.43, 5.31)	0.74
Language 2-year	−2.51 (−6.99, 1.98)	0.27	−1.91 (−6.46, 2.63)	0.41
Motor 2-year	−0.09 (−3.83, 3.64)	0.96	0.52 (−3.44, 4.49)	0.80
Social–emotional 2-year	−4.86 (−10.82, 1.11)	0.11	−3.71 (−9.80, 2.38)	0.23

Bolded *p*–values are statistically significant.

**Figure 2:**
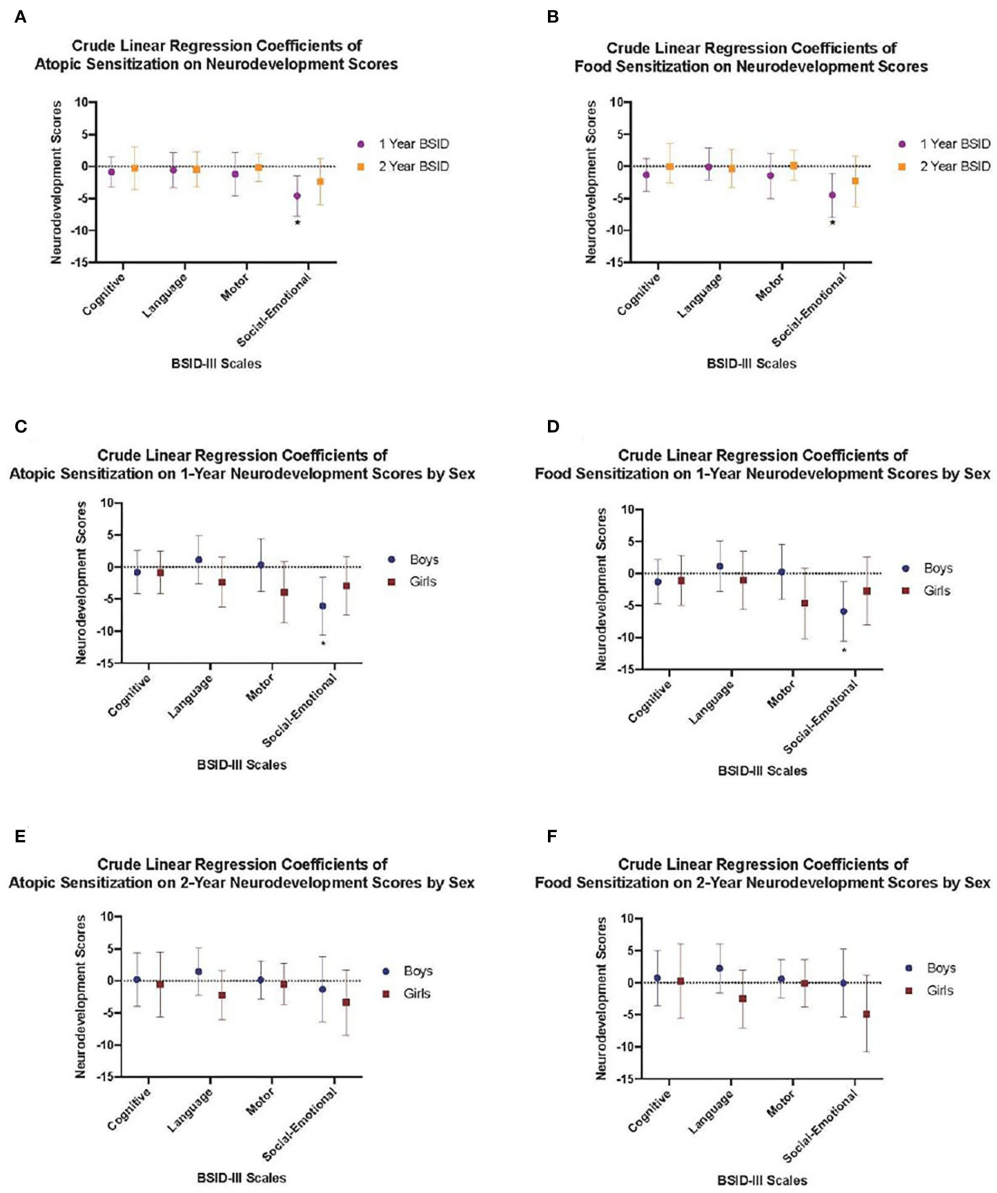
Crude linear regression models demonstrating **(A)** Crude models of atopic sensitization at 1 year on neurodevelopement scores at 1 and 2 years, **(B)** Food sensitization at 1 year on neurodevelopement scores at 1 and 2 years, **(C)** Atopic sensitization at 1 year on neurodevelopement scores at 1 year stratified by child sex, **(D)** Food sensitization at 1 year on neurodevelopement scores at 1 year stratified by child sex, **(E)** Atopic sensitization at 1 year on neurodevelopement scores at years stratified by child sex, and **(F)** Food sensitization at 1 year on neurodevelopement scores at 2 years stratified by child sex. Crude regression coefficient estimates are shown by closed circles and whiskers represent the 95% confidence interval.

Multivariable regression models revealed that the inverse association between 1-year social-emotional scores and food or atopic sensitization remained after adjusting for maternal ethnicity. Specifically, there was a reduction in social-emotional scores among infants who developed atopic (adjusted beta-coefficient: −4.13; 95% CI: −7.41, −0.86) or food sensitization (adjusted beta-coefficient: −4.01; 95% CI: −7.55, −0.47) compared to their infants who did not develop atopic or food sensitization (Figure 2; Table 2). For sensitization to a food or aeroallergen, this association was limited to male infants (adjusted beta-coefficient: −5.22; 95% CI: −9.96, −0.47) and not found among female infants (adjusted beta-coefficient: −2.92; 95% CI: −7.50, 1.66, Figure 2; Table 2). For sensitization to food, the association was also more evident in male infants (adjusted beta-coefficient: −4.85; 95% CI: −9.82,0.11, *p* = 0.06) than in female infants (adjusted beta-coefficient: −2.82; 95%CI: −8.07, 2.43), although it did not reach statistical significance in either sex (Figure 2; Table 2).

In order to strengthen the validity of our results, we conducted a sensitivity analysis for the associations between sensitization and infant neurodevelopment using the standard ≥3-mm SPT cut-off (21). Results from the fully adjusted model using the conventional ≥3-mm wheal size also revealed decreased socio-emotional scores for atopic (adjusted beta-coefficient: −4.99; 95%CI: −8.93, −1.04) and food sensitization (adjusted beta-coefficient: −5.22; 95% CI: −9.50, **−0.9**4). Male infants with atopic sensitization remained more affected than their female counterparts [−5.17 (95% CI: −11.14, −0.80), *p* **=** **0.0**9 vs. adjusted beta-coefficient: −4.21; 95% CI: −9.72, 1.31, respectively]. When the standard ≥3-mm cut-off for sensitization was used, results were consistent with our findings that used the ≥2-mm wheal size — male infants with food sensitization at 1 year had lower, albeit non-significant socio-emotional scores (adjusted beta-coefficient: −4.61; 95% CI: −10.96, 1.74, *p* = 0.154) than female infants (adjusted beta-coefficient: −4.88% CI: −11.08, 1.32).

Since non-normal distributions were noted in all four BSID-III subscales for atopy and food sensitization at 1 year of infant age, we conducted a log transformation of these scores. Our analysis revealed no changes to the significance of the β-coefficients of the transformed variables. For example, the crude socio-emotional scores at 2 years among all the infants were not statistically significant [−2.36 (95% CI: −5.98, 1.27), *p* = 0.2], and the same is observed when it was log-transformed [−0.131 (95% CI: −0.347, −0.085), *p* **=** 0.2]. Again, running the same crude analysis with the ≥3-mm SPT cut-off also led to a nonsignificant association between atopy at 1 year and socio-emotional scores at 2 years [−2.79 (95% CI: −8.29, 2.70), *p* = 0.3] and in a log-transformed model [−0.022 (95% CI: −0.07, −0.03), *p* = 0.4]. Using an SPT cut-off of ≥3 mm did not produce a statistically significant outcome with any of the other Bayley's III neurodevelopmental subscales.

Consequently, this resulted in no changes to our main findings.

Covariate adjustment did not uncover statistically significant associations between infant atopic or food sensitization, and neurodevelopmental outcomes, with one exception: cognitive and language scores at age 2 were higher in male infants with food sensitization (Table 2).

## Discussion

In a general population of 537 Canadian infants, there was no convincing evidence of temporal associations between IgE-mediated atopy or food sensitization status at 1 year and neurodevelopmental outcomes at 2 years of age. These findings concur with the reported absence of association between 12-month atopic or food sensitization and BSID-III neurodevelopment milestones at 18 months in a high-allergy-risk Australian cohort (13), as well as between infant serum IgE levels during the 1st years of life and attention deficit disorders at school age in a general population US cohort (25). Also, we did not find correlational associations between atopy status and neurodevelopment in 1-year-old children, with one exception. Independent of maternal ethnicity, infants with atopic sensitization had reduced scores by 4.13 points (*p* = 0.01) on the social-emotional domain of Bayley's scales compared to their infant counterparts who were not atopic. Similarly, the infants experiencing food sensitization also exhibited 4-point lower social-emotional scores than the infants who did not have food sensitization (*p* = 0.03). However, all of these associations were limited to male infants, such that social-emotional scores were lowered by 5 points if atopic sensitization was present (−5.22 (95% CI: −9.96, −0.47)] and, similarly, if food sensitization was present [−4.85 (95% CI: −9.82, 0.11), *p* = 0.06]. No cross-sectional associations were found in the female infants, and their social-emotional scores were equivalent to that of the male infants. The social-emotional subscale evaluates an infant's interaction, emotionality, self-regulation, and reactivity (26). It is a strong predictor of future behavioral or emotional disorders in childhood and academic achievement in later life (26, 27). We will consider possible bi-directional explanations for our findings in the following paragraphs.

Indeed, the cross-sectional nature of the atopy-socio-emotional development association would not support a causal hypothesis put forward by Chua et al. (10), which points to evidence on temporal associations and common risk factors between atopic conditions and neurodevelopment disorders. The association was not affected by study covariates and possibly acted through factors we did not measure. Mikkelsen et al. (28) reported that food sensitization in infants, for example, to milk, presents challenges and induces stress for new parents as they attempt to feed their infant. Such stressful environments may impact negatively on infant social-emotional development scores. Indeed, this has been reported in preterm and term infants, where studies document the influence of parental postnatal stress or a lack of positive affect on the socio-emotional development of offspring (29, 30). Finally, we observed lower but not statistically significant social-emotional scores at age 2 years in female infants with food sensitization, which appears to be a similar trend to that reported among 1-year-old infants with food allergy in the Australian cohort (13).

An alternate speculation involves reverse causation in which socio-emotional impairment, secondary to a stressful environment, is in the pathway to atopic or food sensitization. Stressors for infants, such as low maternal sensitivity or psychological distress, have been linked to atopic dermatitis (31) and functional gastrointestinal disorders (32). A mother's distress while breastfeeding can alter milk microbiota (33) or lower milk secretory immunoglobulin A (34), both of which affect infant gut immunity (35). Hence, impaired mother-infant feeding or social interactions may lower socio-emotional responses in the infant (36) and, *via* the gut-brain axis, lead to food intolerance (37). Since parent-child interactions, namely strategies for infant soothing, were not assessed in our study, we are unable to offer explanations for the unexpected findings on food sensitization and improved cognition or language in boys. Parent use of strategies, such as cuddling to soothe fussy infants, may change electrocortical rhythms in the infant's brain toward improved neurodevelopmental outcomes (38). A detailed assessment of infant stress responses in studies of atopic disease and neurodevelopmental outcomes is needed to unpack these associations.

Furthermore, we believe that gender bias may also influence our male-specific findings that are worthy of further investigation. Globally, research reporting parental gender bias is becoming well established. For example, a study in Germany revealed that male children of parents who believe girls are better at reading exhibited lower reading-related competence beliefs and were discouraged from reading (39). Consistent with this research, a study in Bangladesh demonstrates that more parents invest in their male children's education and health expenses than their female offsprings (40). Research from Balkan and Scandinavian countries confirms these findings that biased parents allocate greater resources to children of their preferred gender (41). Overall, these studies demonstrate the need to address gender bias in pediatric research as it appears to impair critical areas of child development.

There are several strengths of our study: (i) objective and standard assessment of atopic sensitization in infants with skin prick testing, (ii) objective assessment of neurodevelopment by experts using a well-validated and widely-used standardized measure, (iii) neurodevelopmental assessment at two-time points to enable testing of cross-sectional and temporal associations, and (iv) large sample size in a general population of children that enabled adjustment for early life covariates that were determined from the construction of a direct acyclic graph (DAG). On the other hand, we had no information on whether the study infants with food sensitization had clinically significant signs and symptoms of food allergy, a critical element for proposed hypotheses for our findings. However, their parents would likely have seen the skin wheal reactions to the skin prick testing. We were also unable to examine the infants at high risk for neurodevelopmental morbidity as the CHILD Cohort Study excluded preterm birth below 34 weeks of gestation. Other high-risk groups under-represented in our study were families of low socioeconomic status. Further studies are required to investigate the generalizability of our findings to other populations.

## Conclusion

In our study, atopic and food sensitization at 1 year did not predict neurodevelopmental outcomes at 2 years of age. However, atopic and food sensitization status at 1 year was cross-sectionally associated with reduced social-emotional scores among male infants. We speculated on bidirectional associations that may explain this inverse association. Since mother-infant interactions play a critical role in the socio-emotional development of infants, our study supports the need for additional research on maternal and infant risk factors between atopic and food sensitization and neurodevelopmental disorders. Possible biologic pathways that explain associations between atopic and food sensitization on infant neurodevelopment also merit further evaluation.

## Data availability statement

The raw data supporting the conclusions of this article will be made available by the authors, without undue reservation.

## Ethics statement

The studies involving human participants were reviewed and approved by University of Alberta Health Research Ethics Boards. Written informed consent to participate in this study was provided by the participants' legal guardian/next of kin.

## Author contributions

AK contributed to the conception and design of the study. PM and JP organized the database. NR, CT, and AK wrote the first draft of the manuscript. All authors contributed to the article and approved the submitted version.

## Funding

The Canadian Institutes of Health Research and the Allergy, Genes, and Environment (AllerGen) Network of Centres of Excellence provided initial funding for the CHILD Cohort Study. This research was part of the Edmonton-site Sleep and Neurodevelopment substudy, supported by grant No. 211722 from The Canadian Institutes of Health Research and by the Women and Children's Health Research Institute. These entities had no role in the design and conduct of the study; collection, management, analysis, and interpretation of the data; and preparation, review, or approval of the manuscript.

## Conflict of interest

The authors declare that the research was conducted in the absence of any commercial or financial relationships that could be construed as a potential conflict of interest.

## Publisher's note

All claims expressed in this article are solely those of the authors and do not necessarily represent those of their affiliated organizations, or those of the publisher, the editors and the reviewers. Any product that may be evaluated in this article, or claim that may be made by its manufacturer, is not guaranteed or endorsed by the publisher.
