# Supplementary material for: Sex-specific associations among infant food and atopic sensitizations and infant neurodevelopment
Source: Front Pediatr. 2022 Sep 6;10:734428. doi: 10.3389/fped.2022.734428 (PMC9648178; doi:10.3389/fped.2022.734428)

Table S1. Unique adjustment sets for each multivariable regression model.

Atopic Sensitization Multivariate Model Adjustments

Covariate	Cognitive 1-Year	Language 1-Year	Motor 1-Year	Social-Emotional 1-Year	Cognitive 2-Year	Language 2-Year	Motor 2-Year	Social-Emotional 2-Year
Maternal Age								
Maternal Ethnicity								
Maternal Prenatal Diet Fruit Intake								
Maternal Prenatal Smoking								
Maternal Prenatal Depression								
Maternal Asthma								
Siblings								
Infant Diet (Solids)								
Breastfeeding Duration								
Mode of Birth								
Gestational Age								

Food Sensitization Multivariate Model Adjustments

Covariate	Cognitive 1-Year	Language 1-Year	Motor 1-Year	Social-Emotional 1-Year	Cognitive 2-Year	Language 2-Year	Motor 2-Year	Social-Emotional 2-Year
Maternal Age		■			■	■		
Maternal Ethnicity	■	■	■	■	■	■	■	■
Maternal Prenatal Diet Fruit Intake		■			■	■	■	
Maternal Prenatal Smoking		■	■		■	■	■	
Maternal Prenatal Depression	■	■	■		■	■	■	
Maternal Asthma	■	■			■	■	■	
Siblings		■			■		■	
Infant Diet (Solids)		■	■		■	■	■	
Breastfeeding Duration		■			■	■	■	
Mode of Birth	■	■	■		■		■	■
Gestational Age	■	■	■		■	■	■	

Note: Variables identified as potential covariates from the DAG (Figure 1) were individually tested for a greater than 15% change to the estimate. Only covariates that were identified in both the DAG and caused a greater than 10% change to the estimate (shown in pink) were added to the minimal adjustment set for the corresponding multivariable model.

Table S2. Frequency characteristics for categorical variables in the study sample of infants with atopic and food sensitization at 1 year and neurodevelopmental data at 1 and 2 years of age (n=537)

Maternal characteristics	Total N	n (%)	Infant characteristics	Total N	n (%)
**Family Income**	488		**Atopic Sensitization**	537	
Less than 39,999		26 (5.3)	Yes		88 (16.4)
40,000 to 79,999		121 (24.8)	No		449 (83.6)
80,000 to 99,999		79 (16.2)	**Food Sensitization**	537	
Exceeds 100,000		262 (53.7)	Yes		72 (13.4)
**Maternal Education**	515		No		456 (86.6)
Some/finished high school		36 (7.0)	**Child Sex**	537	
Some university/college		193 (37.5)	Boys		279 (52.0)
University degree		286 (55.5)	Girls		258 (48.0)
**Maternal Asthma**	519		**Breastfeeding 3 Months**	535	
Yes		123 (23.7)	None		77 (14.4)
No		396 (76.7)	Partial		146 (27.3)
**Prenatal Smoking**	519		Exclusive		312 (58.4)
Yes		19 (3.7)	**Birthmode**	532	
No		500 (96.3)	Vaginal no IAP		277 (52.1)
**Maternal Depression**	483		Vaginal IAP		127 (23.9)
Yes		96 (19.9)	CS-Elective		58 (10.9)
No		387 (80.1)	CS-Emergency		70 (13.2)
**Maternal Age**	537		**Gestational Age**	537	
18-29		150 (27.9)	37 weeks+		506 (5.8)
30-39		366 (68.2)	34-36 weeks		31 (5.6)
40+		21 (3.9)	**Siblings**	535	
**Maternal Ethnicity**	533		Yes		302 (56.5)
White Caucasian		420 (78.8)	No		233 (43.5)
Asian		51 (9.6)			
Other		62 (11.6)			

Table S3. Frequency characteristics for continuous variables in the study sample of infants with atopic and food sensitization at 1 year and neurodevelopmental data at 1 and 2 years of age (n=537)

Continuous variables	Total N	Mean (SD)	Min	Max
**BSID-III Cognitive 1 Year**	537	110.04 (10.3)	75	145
Missing, n (%)	0 (0)			
**BSID-III Language 1 Year**	536	107.9 (11.9)	65	147
Missing, n (%)	1 (0.2)			
**BSID-III Motor 1 Year**	535	102.8 (13.6)	70	154
missing, n (%)	2 (0.4)			
**BSID-III Social-Emotional 1 Year**	519	102.6 (13.8)	60	145
Missing, n (%)	18 (3)			
**BSID-III Cognitive 2 Year**	537	105.7 (14.3)	70	145
Missing, n (%)	0 (0)			
**BSID-III Language 2 Year**	536	100.2 (12.0)	68	135
Missing, n (%)	1 (0.2)			
**BSID-III Motor 2 Year**	537	98.9 (9.5)	67	127
Missing, n (%)	0 (0.0)			
**BSID-III Social-Emotional 2 Year**	527	108.7 (15.7)	60	145
Missing, n (%)	10 (1.9)			
**Maternal Pregnancy Fruit Intake**	508	3.2 (2.0)	0.14	13.4
Missing, n (%)	29 (5.4)			
**Breastfeeding Duration**	517	10.2 (6.7)	0	25
Missing, n (%)	10.2 (6.7)			

Table S4. Percentage distribution of food and atopic sensitization at 1 year across candidate covariates (n=537)

Categorical variables	Atopic Sensitization 1YR (**YES**) (16.4% overall) N[c] (%)	Atopic Sensitization on 1YR (**NO**) (83.6% overall) N[c] (%)	p-value	Food sensitization (**YES**) (13.4% overall) N[c] (%)	Sensitization on 1YR (**NO**) (86.6%) N[c] (%)	p-value
Maternal Characteristics						
**CESD**			0.386[a]			0.553[a]
Never	77 (16.8)	382 (83.2)		64 (13.9)	395 (86.1)	
Prenatal	7 (23.6)	24 (77.4)		5 (16.1)	26 (83.9)	
Postnatal	4 (10.3)	35 (89.7)		3 (7.7)	36 (92.3)	
Persistent	0 (0.0)	8 (100)		0 (0.0)	8 (100.0)	
**Maternal age**			0.638[a]			0.294[a]
18 to 29	21 (14.0)	129 (86.0)		15 (10.0)	135 (90.0)	
30 to 39	64 (17.6)	302 (82.5)		55 (15.0)	311 (85.0)	
Over 40	3 (14.3)	18 (85.7)		2 (9.5)	19 (90.5)	
**Maternal education**			**0.038[a]**			**0.010[a]**
Some/finished high school	4 (11.1)	32 (88.9)		3 (8.3)	33 (91.7)	
Some university/ college	23 (11.9)	170 (88.1)		16 (8.3)	177 (91.7)	
University degree	58 (20.3)	228 (79.7)		50 (17.5)	236 (82.5)	
**Prenatal smoking**			0.223[a]			0.091[a]
Yes	1 (5.3)	18 (94.7)		0 (0.0)	19 (100.0)	
No	86 (17.2)	414 (82.8)		71 (14.2)	429 (85.8)	
**Maternal ethnicity**			**<0.001**			**<0.001**
White Caucasian	53 (12.6)	367 (87.4)		43 (10.2)	377 (89.8)	
Asian	19 (37.3)	32 (62.8)		15 (29.4)	36 (70.6)	
Other	15 (24.2)	47 (75.8)		13 (21.0)	49 (79.0)	
**Maternal asthma**			0.838			0.822
Yes	20 (23.0)	95 (22.0)		15 (13.0)	100 (87.0)	
No	67 (77.0)	337 (83.4)		56 (12.9)	348 (86.1)	
Infant characteristics						
**Child sex**			0.595			0.095
Boys	48 (17.2)	231 (82.8)		44 (15.8)	235 (84.2)	
Girls	40 (15.5)	218 (84.5)		28 (10.9)	230 (89.2)	
**Older siblings**			0.529			0.236

Yes	47 (53.4)	255 (57.1)		36 (15.5)	266 (88.1)	
No	41 (46.6)	192 (82.4)		36 (11.9)	197 (84.6)	
**Birth mode**			0.593			0.958
Vaginal-noIAP	43 (15.5)	234 (84.5)		37 (13.4)	240 (86.6)	
Vaginal-IAP	19 (15.0)	108 (85.0)		15 (11.8)	112 (88.2)	
CS-elective	13 (22.4)	45 (77.6)		8 (13.8)	50 (86.2)	
CS-emergency	11 (15.7)	59 (84.3)		10 (14.3)	60 (85.7)	
**Infant diet - solids at 3M**			0.704			1.000[a]
Yes	1 (7.7)	12 (92.3)		1 (7.7)	12 (92.3)	
No	86 (16.6)	432 (83.4)		70 (13.5)	448 (86.5)	
**Infant breastfeeding duration (months)**			0.220			0.200
	85(16.4%)	432 (83.6%)		70 (13.54%)	447 (86.46%)	
**Maternal prenatal fruit intake**			0.086			0.059
	83 (16.34%)	425 (83.66%)		68 (13.39%)	440 (86.61%)	
**Gestational age (in weeks)**						
	88 (16.54%)	444 (83.46%)	0.097	72 (13.53%)	460 (86.47%)	0.1713

[a]Fisher's exact test
[b]Bold values are statistically significant
[c]Total number of observations (N) is based per column per atopy/food sensitizatio yes/no

Table S5. Distribution of 1-year BSID-III neurodevelopment subscale scores across candidate study covariates (N=537)

Covariates		Neurodevelopmental Scores 1YR							
		Cognitive Score		Language Score		Motor Score		Social Emotional Score	
	N (%)	Mean (*SD*)	*P* Value	Mean (*SD*)	*P* Value	Mean(*SD*)	*P* Value	Mean (*SD*)	*P* Value
Maternal Categorical factors - Mean (SD)[a]									
**CESD**			0.463		0.739		0.470		0.409
Never	459 (85.5)	110.03(10.20)		107.85 (12.18)		102.64 (14.71)		102.99 (13.96)	
Prenatal	31 (5.8)	108.23 (11.07)		106.10 (10.59)		99.13 (13.40)		99.35 (10.23)	
Postnatal	39 (7.3)	110.77 (9.97)		109.33 (10.95)		104.38 (15.82)		101.43 (14.78)	
Persistent	8 (1.5)	114.38 (14.50)		107.88 (10.26)		105 (9.09)		98.57 (15.20)	
**Maternal Age**			0.595		0.118		0.118		0.595
18 to 29	150 (27.9)	109.35 (9.79)		107.94 (12.16)		103.74 (13.52)		103.60 (14.65)	
30 to 39	366 (68.2)	110.40 (10.46)		107.90 (11.93)		101.85 (15.20)		102.22 (13.54)	
Over 40	21 (3.9)	108.67 (11.24)		106.43 (11.71)		107.57 (11.45)		102.25 (13.33)	
**Prenatal smoking**			0.783		0.039		0.275		0.748
Yes	19 (3.7)	110.79 (9.02)		113.53 (11.03)		106.05 (13.75)		101.39 (10.12)	
No	500 (96.3)	110.12 (10.36)		107.69 (12.13)		102.32 (14.66)		102.45 (13.87)	
**Maternal ethnicity**			0.001		0.001		0.137		0.007
White Caucasian	420 (78.8)	110.47 (9.99)		108.71 (11.80)		102.66 (15.15)		103.13 (13.64)	
Asian	51 (9.6)	105.10 (11.02)		101.94 (13.01)		99.29 (13.50)		99.26 (16.05)	
Other	62 (11.6)	111.21 (11.08)		106.92 (11.31)		104.81 (12.06)		101.55 (13.19)	
Infant Categorical factors - Mean (SD)[a]									
**Child Sex**			0.217		0.000		0.436		0.406
Boys	279 (52.0)	109.51 (10.73)		106.08 (12.04)		102.13 (14.25)		103.09 (14.29)	

Girls	258 (48.0)	110.61 (9.78)		109.76 (11.62)		103.12 (15.09)		102.08 (13.34)	
**Breastfeeding at 3 months**			0.549		0.278		0.265		0.503
Exclusive	312 (58.3)	109.29 (9.79)		107.25 (11.78)		101.49 (11.71)		100.97 (14.88)	
Partial	146 (27.3)	109.60 (11.34)		106.74 (13.28)		101.32 (17.52)		102.53 (13.27)	
None	77 (14.4)	110.46 (9.94)		108.57 (11.37)		103.47 (13.84)		103.07 (13.86)	
**Birth mode**									
Vaginal-no IAP	277 (52.1)	110.56 (10.58)	0.342	107.98 (12.50)	0.881	101.79 (15.74)	0.099	102.90 (13.88)	0.943
Vaginal-IAP	127 (23.9)	110.16 (10.35)		107.83 (10.57)		102.61 (13.58)		101.97 (13.12)	
CS-elective	58 (10.9)	108.79 (8.90)		106.71 (13.21)		100.78 (13.73)		102.68 (15.43)	
CS-emergency	70 (13.2)	108.39 (10.36)		108.31 (11.35)		106.33 (11.85)		102.46 (14.00)	
**Solids**			0.144		0.115		0.864		0.111
No	518 (97.6)	109.40 (10.36)		108.33 (11.76)		102.79 (14.39)		100.85 (13.01)	
Yes	13 (2.4)	110.50 (10.27)		107.53 (12.13)		102.43 (14.91)		103.91 (14.34)	
**Older siblings**			0.220		0.443		0.779		0.012
No	233 (43.6)	109.40 (10.36)		107.53 (12.13)		102.79 (14.39)		100.85 (13.01)	
Yes	302 (56.4)	110.50 (10.27)		108.13 (12.25)		102.43 (14.91)		103.91 (14.34)	
Neurodevelopmental Outcomes – or β (95% CI)[b]									
**Breastfeeding duration**	10.16 (6.69)[c]	0.18 (0.05, 0.31)	0.007	0.08 (-0.069, 0.24)	0.281	0.14 (-0.05, 0.32)	0.157	0.21 (0.03, 0.39)	0.023
**Maternal prenatal fruit intake**	3.16 (1.99)[c]	0.14 (-0.30, 0.59)	0.531	0.263 (-0.26, 0.79)	0.326	0.03(-0.61, 0.67)	0.924	0.57(-0.04, 1.17)	0.068
**Gestational age**	39.11 (1.39)[c]	1.13 (0.50, 1.76)	0.000	0.67 (-0.05, 1.42)	0.067	1.05 (0.15,1.95)	0.022	0.59(-0.28, 1.46)	0.182

Note: BISD-III=Bayley Infant Scales of Development Third Edition; SD=standard deviation; *β=Coefficient.* Total number of observations (N) is based per column covariate and it's corresponding categories

[a]Analyzed by *t*test or one-way analysis of variance, [b]Analyzed by linear regression, [c] Reported in means (standard deviation). Gestational age is measured in weeks and maternal prenatal fruit intake assessed using the Healthy Eating Index (HEI)

Table S6. Distribution of 2-year BSID-III neurodevelopment subscale scores across candidate study covariates (N=537)

Covariates		Neurodevelopmental Scores 2YR							
		Cognitive Score		Language Score		Motor Score		Social Emotional Score	
	N (%)	Mean (*SD*)	*P* Value	Mean (*SD*)	*P* Value	Mean (*SD*)	*P* Value	Mean (*SD*)	*P* Value
Maternal Categorical factors - Mean (SD)[a]									
**CESD**									
Never	459 (85.5)	106.09 (14.42)	0.364	100.40 (11.86)	0.105	99.03 (9.45)	0.915	109.34(15.73)	0.015
Prenatal	31 (5.8)	104.52 (13.87)		100.84 (12.88)		98.13 (10.64)		102.67 (14.49)	
Postnatal	39 (7.3)	104.23 (14.80)		98.90 (13.22)		98.26 (8.77)		108.29 (15.65)	
Persistent	8 (1.5)	98.13 (5.94)		90.25 (9.45)		98.13 (11.89)		95.01 (11.55)	
**Maternal Age**									
18 to 29	150 (27.9)	107.33 (15.12)	0.226	101.52 (11.35)	0.153	99.65 (9.27)	0.512	109.18 (15.18)	0.856
30 to 39	366 (68.2)	105.02 (13.94)		99.48 (12.24)		98.59 (9.64)		108.57 (16.04)	
Over 40	21 (3.9)	107.14 (15.13)		102.33 (12.60)		99.10 (8.50)		107.38 (14.72)	
**Prenatal smoking**			0.135		0.358		0.664		0.274
Yes	19 (3.7)	100.79 (13.15)		97.63 (9.62)		98 (8.02)		104.74 (16.79)	
No	500 (96.3)	105.77 (14.30)		100.23 (12.15)		98.98 (9.66)		108.76 (15.67)	
**Maternal ethnicity**									
White Caucasian	420 (78.8)	106.90 (14.41)	0.002	101.45 (11.39)	0.000	98.98 (9.33)	0.528	109.40 (15.77)	0.201
Asian	51 (9.6)	101.27 (13.30)		93.02 (13.31)		97.53 (10.35)		105.60 (15.41)	
Other	62 (11.6)	101.81 (13.77)		97.63 (12.93)		99.44 (10.19)		107.25 (15.42)	
Infant Categorical factors - Mean (SD)[a]									

**Child Sex**									
Boys	279 (52.0)	103.36 (13.41)	0.000	97.18 (11.86)	0.000	98.02 (9.45)	0.024	107.26 (16.14)	0.031
Girls	258 (48.0)	108.33 (14.88)		103.41 (11.38)		99.87 (9.46)		110.22 (15.16)	
**Breastfeeding at 3 months**			0.018		0.002		0.283		0.119
Exclusive	312 (58.1)	102.86 (10.71)		98.05 (10.48)		97 .66(8.29)		107.40 (17.90)	
Partial	146 (27.2)	104.25 (13.23)		97.94 (11.13)		98.4 (9.45)		106.82 (16.01)	
None	77 (14.3)	107.23 (15.45)		101.71 (12.56)		99.4 (9.80)		109.87 (14.92)	
**Birth mode**			0.068		0.082		0.246		0.760
Vaginal-no IAP	277 (52.1)	107.03 (15.14)		101.12 (11.16)		99.58 (9.01)		109.10 (16.34)	
Vaginal-IAP	127 (23.9)	104.53 (12.35)		100.08 (12.67)		98.29 (9.14)		108.17 (14.39)	
CS-elective	58 (10.9)	101.98 (14.23)		96.78 (13.18)		97.05 (10.87)		107.02 (16.20)	
CS-emergency	70 (13.2)	105.86 (14.17)		99.26 (12.99)		98.91 (10.49)		109.57 (15.40)	
**Solids**			0.423		0.386		0.652		0.674
No	518 (97.6)	105.93 (14.47)		100.31 (11.93)		98.97 (9.42)		108.79 (15.52)	
Yes	13 (2.4)	102.69 (9.04)		97.38 (15.63)		97.77 (12.62)		106.92 (23.68)	
**Older siblings**			0.931		0.596		0.005		0.462
No	233 (43.6)	105.64 (14.73)		100.44 (12.00)		97.58 (9.33)		108.07 (15.52)	
Yes	302 (56.4)	105.75 (14.05)		99.88 (12.08)		99.91 (9.51)		109.08 (15.90)	
Neurodevelopmental Outcomes – or β (95% CI)[b]									
**Breastfeeding Duration**	10.16 (6.69)[c]	0.26 (0.08, 0.44)	0.006	0.30 (0.14, 0.45)	0.000	0.07 (-0.05, 0.20)	0.237	0.20 (-0.007, 0.40)	0.059
**Maternal prenatal fruit intake**	3.16 (1.99)[c]	-0.39 (-1.01, 0.23)	0.217	0.39 (-0.13, 0.92)	0.144	0.17 (-0.25, 0.59)	0.426	0.54 (-0.15, 1.23)	0.124
**Gestational age**	39.11 (1.39)[c]	1.12 (0.25, 2.00)	0.012	1.13 (0.40, 1.86)	0.002	0.82(0.24, 1.40)	0.006	0.83 (-0.14, 1.81)	0.092

Note: BISD-III=Bayley Infant Scales of Development Third Edition; SD=standard deviation; *β=Coefficient.* Total number of observations (N) represents those participants with maternal distress data and cognitive data collected at the 1-year study visit

[a]Analyzed by *t*test or one-way analysis of variance. [b]Analyzed by linear regression, [c] Reported in means (standard deviation). Gestational age is measured in weeks and maternal prenatal fruit intake assessed using the Healthy Eating Index (HEI)

Figure S1. Residuals resulting from regressing neurodevelopmental scores at 1 year (A-D) and 2 years (E-H) of infant age against infant atopic sensitization status.
Note: S_{kw} = skewness; S_{ku} = kurtosis; p = p value

A.

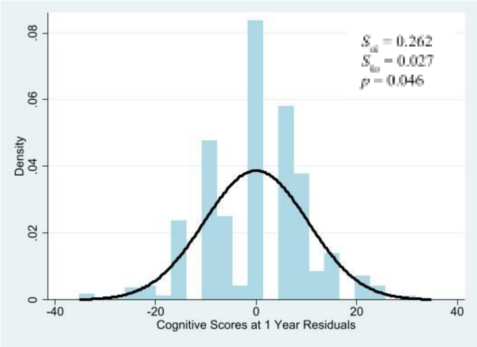

B.

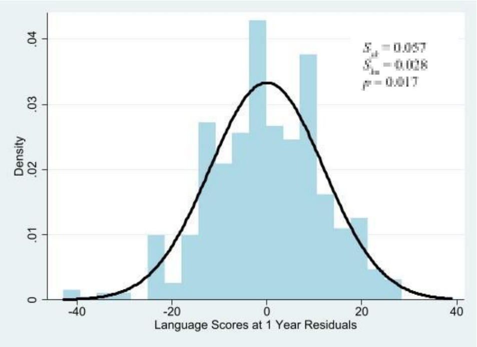

C.

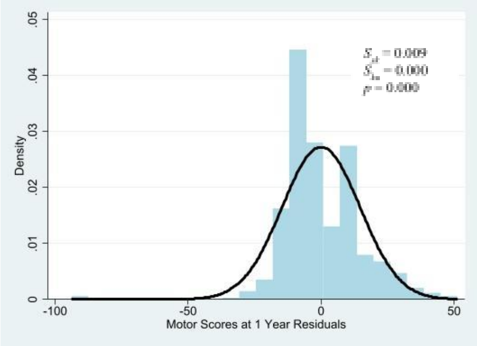

D.

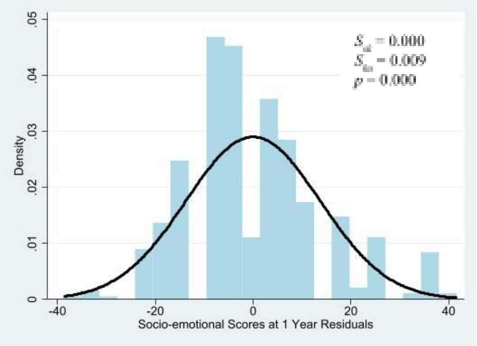

E.

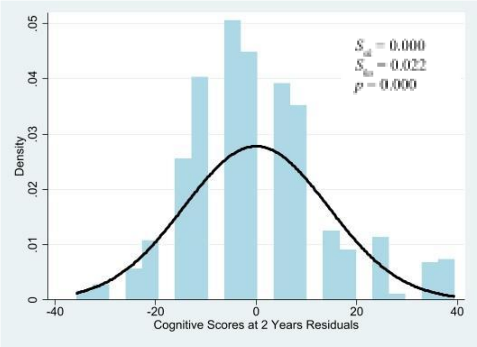

F.

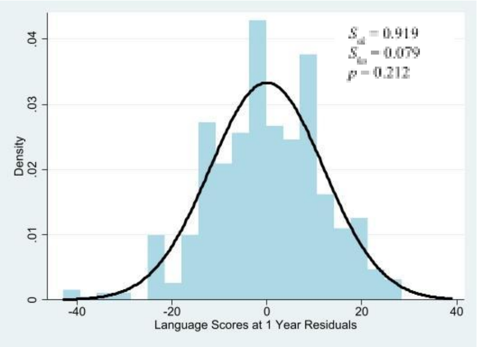

G.

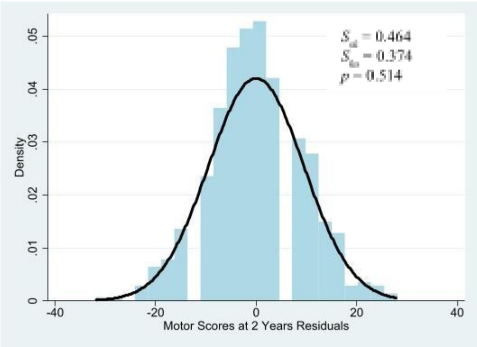

H.

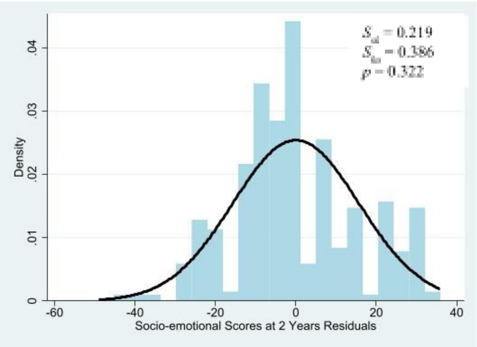

Figure S2. Residuals resulting from regressing neurodevelopmental scores at 1 year (A-D) and 2 years (E-H) of infant age against infant food sensitization status.

Note: S_{kw} = skewness; S_{ku} = kurtosis; p = p value

A.

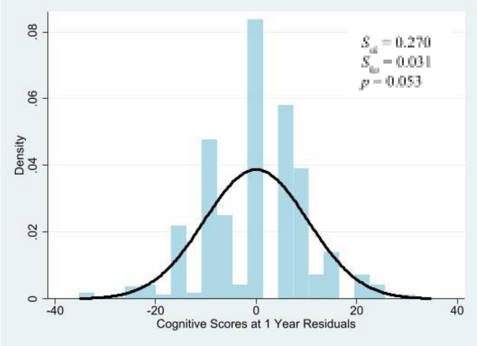

B.

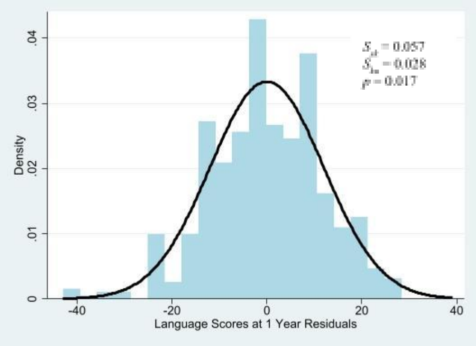

C.

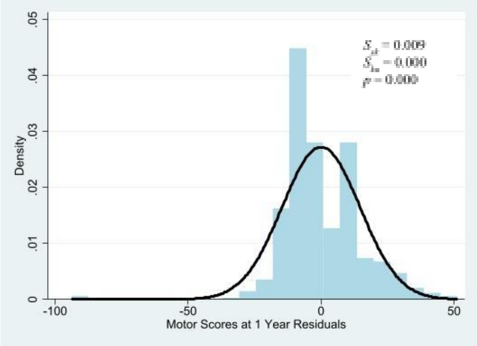

D.

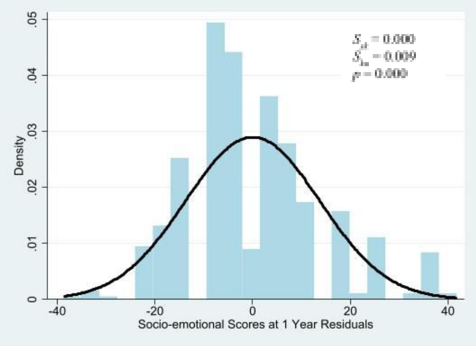

E.

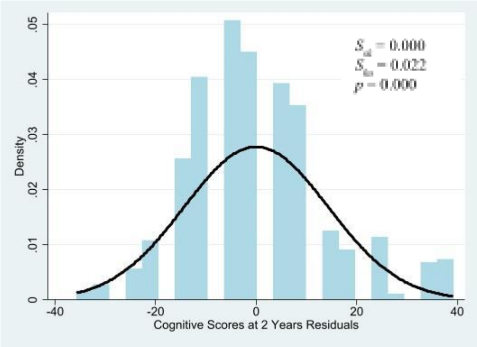

F.

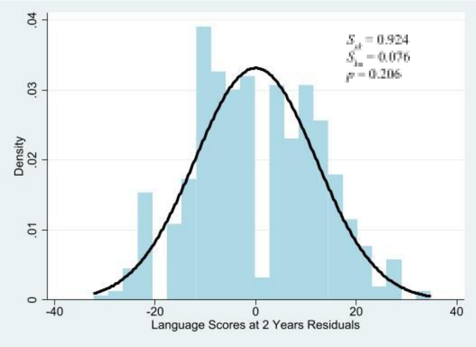

G.

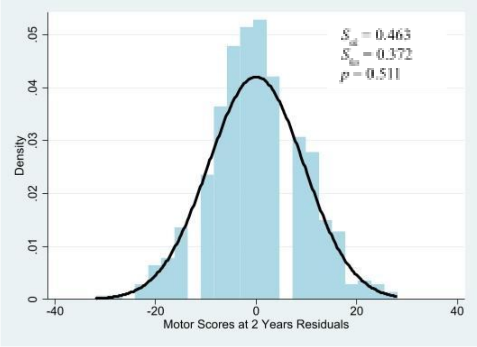

H.

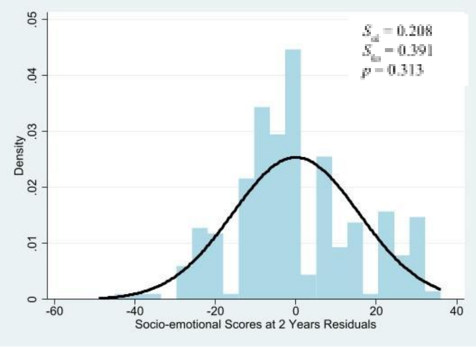

Supplement: Supplementary file 1 [file Data_Sheet_1.PDF]
